# Impact of Weather on Pedestrians’ Slip Risk

**DOI:** 10.3390/ijerph19053007

**Published:** 2022-03-04

**Authors:** Marjo Hippi, Markku Kangas

**Affiliations:** Meteorological Research, Finnish Meteorological Institute, 00560 Helsinki, Finland; markku.kangas@fmi.fi

**Keywords:** pedestrians, weather, road safety, slipperiness, slip, walking, commuting accident

## Abstract

Pedestrians’ slipping injuries are a very typical problem in the Nordic countries, causing varying degrees of injuries and in the worst case, long sick leaves. There is a clear seasonal variation in the number of slips. Sidewalk slipperiness and the risk of slips is a complex combination of weather, winter maintenance activities, number of walkers, and the grip between shoes and surface, as well as human behavioral and physical factors. In this study, the effect of weather on pedestrians’ slipping injuries is studied. Daily weather observations are compared to the slip statistics that have been collected from commuting accident statistics in cases where the way of commuting has been walking. A total of 16 cities from Finland for 14 winters are included in this study. The results reveal that snow on the ground increases the slip risk more than three times compared to no-snow situations. Near zero temperatures and precipitation are very typical on days when slip injuries occur more than usual. However, there are also days when high amounts of slips cannot be explained with the weather. The study also shows that there are significant differences as to the number and timing of slips between different parts of the country.

## 1. Introduction

The Ministry of Transport and Communications has set a programme to promote sustainable transport modes in Finnish municipalities [1]. The aim is to increase trips made on foot or by bicycle, promote public health, improve traffic safety, and decrease the greenhouse gas emissions from transport as well as emissions harmful to air quality [1,2]. The goal is to replace private motoring with sustainable transport modes, especially in urban areas within short distances [3]. The key idea is to make walking and cycling safe, fluent, and attractive as well as to invest in winter maintenance and infrastructure of sidewalks [3]. Similar sustainable transport promotion plans have been made also in many other countries [4].

Pedestrians’ wintertime slipping and falling injuries are a very typical problem for the Nordic society, causing at worst long sick leaves and varying degrees of injuries, like bruises, sprains, and fractures [5,6,7,8,9,10,11,12]. The most common injuries due to falls and slipping are fractures of the wrists and ankles, concussions, and other head injuries [13,14]. However, there seem to be some differences between the age and gender as fractures resulting from slipping among females aged 50 and older seem to be significantly more frequent than among males in the same age range [9]. Slips can be considered a major public health and economic problem [15].

Slips are due to fairly complex causal pathways, involving both environmental and human factors [16]. Sidewalk slipperiness and risk of slips is a combination of weather, winter maintenance activities, number of walkers, and grip between shoes and surface as well as human behavioral and physical factors. Many of these factors are difficult, or even impossible, to consider when studying the slipping risk. Weather is the only factor studied in this article. Other factors can cause uncertainty to the results.

There is a clear seasonal variation in the number of slips, as more slips occur in the wintertime compared to summertime. Weather has a significant effect on the amount of slipping, as snow and ice increase the risk of slipping. Slipping and falling accidents and injuries occurring when there is snow or ice on the ground have been reported in the Nordic and European countries and in Japan [8,12,17,18,19,20,21]. Slips cause congestion for rescue centers and healthcare especially on the most slippery days. The emergency clinics of the hospitals can be crowded, and surgery queues grow due to high slip rates.

Accidents and injuries occurring to pedestrians and cyclists are typically highly underreported as they are often single accidents (thus, without a collision with another party) and are not included in traffic accident statistics [22,23]. This is a problem not only in Finland but also in many other countries [24,25]. No complete statistics about slips and falls exist. However, the information about pedestrians’ slip and fall amounts can be estimated from other data sources, like the Finnish care register for Health Care (HILMO), ambulance transport, injury claim data, or from other injury databases of individual companies [26,27].

In Finland, almost every second person slips or falls outdoors annually, and around 70,000 persons (including pedestrians and cyclists) are injured needing medical attention [13]. Slip and fall injuries cause high financial costs each year, with an estimated annual cost of more than 2 billion euros (2006 value) including the costs of medical care, loss of work input and reduced well-being [28]. Reduced well-being is the largest part of the sum covering about 95% of the total costs.

Weather phenomena like freezing, melting, condensation, and precipitation in all forms can cause slipperiness. Slipperiness can also be formed by mechanical ways, when pedestrians or cars compress the snow or plough machines polish the snowy surface into a dense, almost ice-like surface [29,30].

In a previous study [15], three explanatory factors were found for the increased risk of slipping:

1. The daily average temperature was between −2 and 0 °C;
2. The temperature crossed zero degrees during the day;
3. There was at least some precipitation during the day.

Also, a rapid drop in temperature appeared to increase the number of slips.

Finnish Meteorological Institute (FMI) has developed a numerical model, RoadSurf-Pedestrian, that predicts the state of slipperiness on the sidewalks due to weather [30]. FMI also issues warnings for the public in case when very slippery sidewalk conditions are forecasted. RoadSurf-Pedestrian classifies the expected sidewalk slipperiness into three classes: normal, slippery, and very slippery. Very slippery cases are divided into four categories: slipperiness due to packed snow, freezing, snow above ice and water above ice. RoadSurf-Pedestrian is used by meteorologists when issuing the warning of challenging or dangerous sidewalk conditions.

This study presents the temporal and spatial statistics of slip amounts and weather parameters when the daily number of slips is high. Also, the number of injuries on winter days and non-winter days are compared. The slip injury data is based on insurance data covering the injuries occurring to people while commuting. Data is provided by the Finnish Workers’ Compensation Center (TVK). A total of 16 cities around Finland are selected for this study. The analyses are performed using the TVK’s slip injury data (people on the working age) regardless of age and sex. Weather data is based on daily weather observations of daily temperature (mean, minimum, maximum), precipitation, and snow amount. Time period for the study is between 1 October 2005, and 30 September 2019.

The results of this study yield more detailed information and better understanding about the weather phenomena causing slipperiness and days with a high number of slips and falls. The global warming and its effect on slip risk are speculated as well. The findings can also be used to improve the RoadSurf-Pedestrian sidewalk condition model.

## 2. Materials and Methods

In the present study, the slipping injury data is analyzed and compared to weather data. This data, obtained from the TVK, is used as an indicator of slippery conditions on sidewalks. TVK coordinates the practical application of workers’ compensation. In Finland, the employers are obliged to insure their employees against work related accidents and injuries. The analyzed data include injuries that have occurred while commuting and for which the insurance company has paid compensation from the occupational accident insurance. TVK’s injury data are based on self-reported crashes handled by the insurance companies that have paid the compensation [23]. In this paper, a single-pedestrian injury is determined as an event when a pedestrian has slipped, fallen, or stumbled. Henceforth, slipping includes falls and trips when examining the TVK’s slipping, falling, and stumbling injuries.

The data includes slips on daily and municipal level; there is no specific information available of the time or area the slip occurred. In this study, TVK’s data doesn’t include information about the type or severity of the injury or the socio-demographic characteristics of the victims. Even though the TVK’s slip injury data doesn’t cover all of the slip injuries that occur in Finland, it gives enough information about the daily slip amounts, especially when the daily number of slip injuries have been high. Also, variation on slip amounts between the days can be seen. If the number of the daily slips is high, it is assumed here that the weather will affect slipperiness and slip amounts.

Nine percent of all commuting trips in Finland are made entirely on foot [31]. Walking is more common in bigger cities than in smaller cities [31]. However, all road users are pedestrians at some point of their journey [32], as the driver or passenger of a vehicle is interpreted as a pedestrian in the case of slipping or falling after leaving the vehicle. The slip-related injuries occur typically on the sidewalk, outdoor path, courtyard or in a parking lot [13,28,33]. Slipping occurs often when unexpected sudden loss of grip is encountered [34].

Accidents are typically assumed to be Poisson-distributed as they typically occur randomly and independently [35]. The peak day of pedestrians’ slipping injuries is determined as a day when the number of slipping injuries exceeds the threshold of the number with probability less than 0.01% in a Poisson distribution [15,36]. Similarly, a potential peak day with increased risk of slipping is defined with a probability less than 1%. It can be assumed that the slipping risk is higher when sidewalks are slippery due to weather.

The thresholds for a peak day and a potential peak day are defined separately for all cities in this study. Typically, the more citizens with a high number of total slips, the higher the peak day definition threshold. The daily peak day threshold varies between 6 and 25, and potential peak day between 4 and 18, as presented in Table 1. The daily peak day threshold for a specific city is the same for all winter seasons, thus revealing the changes in slip amounts between the years and months. Threshold values are calculated using the total slip amount values for the whole period between 2005 and 2019.

The daily weather observations are studied and compared to the daily slipping injury amounts. Of special interest are days when the number of slipping injuries is high (the so-called peak days of slipping injuries). Weather observations on the days preceding the peak days of slipping injuries are also studied. The focus of this study is to find the weather situations that are dominant and that use to occur when the daily slip amount is high. Mean temperatures, zero crossings, precipitation and snow amount are studied as well as temporal and areal differences. Other factors, like the amount of public transport, the mode of transport while commuting, and the length of the work trip are not considered in this study.

**Figure 1:**
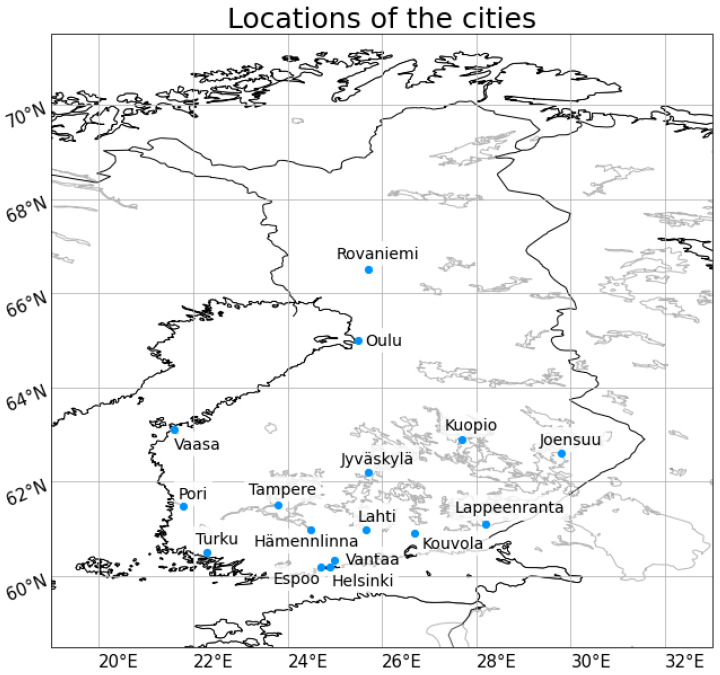
Location of the cities presented on the map.

A total of 16 cities have been selected for this study (see Figure 1 and Table 1). According to the TVK’s data, the selected cities are among the 16 cities in Finland with the highest yearly slip amounts. In terms of population, the cities are the 15 largest ones in Finland, with Rovaniemi being the 17th largest. Places are located around Finland, thus representing different climatological areas from coastal areas to inland and from south to north. Helsinki, Espoo, Vantaa, Turku, Pori, Vaasa, and Oulu are located in the coastal area and the sea has a strong impact on the weather. Oulu is located further north and due to its northern location, it is colder and there is more sea ice in winter than in other coastal cities. Joensuu and Kuopio are the most continental sites having the longest distance to the sea, whereas Rovaniemi is located in the northernmost and climatologically coldest place. Other cities, Lahti, Kouvola, Hämeenlinna, Tampere, Jyväskylä and Lappeenranta, are located inland.

The number of slips differs much between the cities and is strongly related to the number of citizens or employees (see Table 1). The results are statistically more reliable for bigger cities compared to smaller cities where the amount of daily slipping injuries can be quite low. Forms of transportation to work (car, biking, walking, or the use of public transportation), the amount of public transportation, the number of pedestrians, and the winter maintenance rules may vary between the cities, but that information has not been considered in this study. Table 1 also presents the total and wintertime slip amounts, slip injury rate per 1000 employees, number of peak days (including potential peak days), and the calculated thresholds for peak and potential peak day, separately for each city.

Slips occurring on weekends and public holidays are highly underestimated in the TVK’s slip injury data as people during these days work less than on normal weekdays. Also, days around public holidays, like Christmas, New Year and Easter as well as the typical winter holiday season in the end of February and early March cause slight uncertainty to the results with reduced amounts of daily slipping amounts, as people are not working, and community accidents don’t occur that much compared to normal weekdays.

As meteorological data, daily observations that were calculated as spatial averages for the study areas from the gridded temperature data set on the spatial resolution of 10 km × 10 km, were used. Daily weather observations include information of temperature (daily mean, minimum, maximum), daily precipitation sum, and snow depth. The daily mean temperature is calculated as the mean of the eight daily synoptic measurements. The daily minimum and maximum temperatures are measured between 18 UTC on the previous day and at 18 UTC on the date of the observation. Daily precipitation sum is recorded between 6 UTC on the date of the observation and 6 UTC on the following day, snow depth is recorded on 6 UTC. The data is produced operationally at the FMI from the station-wise temperature observations by Kriging interpolation that also takes into account the elevation and the share of water bodies [38,39].

As weather observations are available only on a daily level, there is no information for example about the timing of weather phenomena, like zero crossings, temperature variation or precipitation. Also, the precipitation form is not available. In addition, it is not known whether the sidewalk surface is covered by ice or snow or not. Thus, the snow amount measurements are the best available estimation for the potential icy and snowy sidewalk condition, even though it does not necessarily correlate with the slipperiness for example at the end of the winter season when snow has melted but there is still ice on the sidewalks.

## 3. Results

### 3.1. General Information about Data and Slips

Data between 1 October 2005, and 30 September 2019, is included in this study, covering a total 14 winter seasons. Months from October to April have been defined as winter months. Weather differs a lot during this time, with winters ranging from those with mild temperatures and low snowfall to colder and snowy ones. Also, during a specific winter there can be huge differences between the cities: weather on the coastal areas can be mild with small amounts of snow whereas at the same time, northern and eastern parts of Finland are colder and snowy.

As Table 1 shows, the number of slip injuries varies a lot between the cities: the more citizens (or employees in this table), the more slip injuries. The slip injury rate per 1000 employees is highest in Helsinki (5.3) and lowest in Vaasa (2.9).

Days with increased risk of slipping are considered in this study. They are defined as days when the daily number of slip injuries is equal or greater than the potential peak day threshold. The thresholds of the daily peak days and potential peak days are presented on Table 1. Hereafter, when mentioning peak days, the potential peak days are included as well. The data from all 16 cities include a total of 2549 peak days.

### 3.2. Temporal Distribution of Peak Days of Slipping Injuries

**Figure 2:**
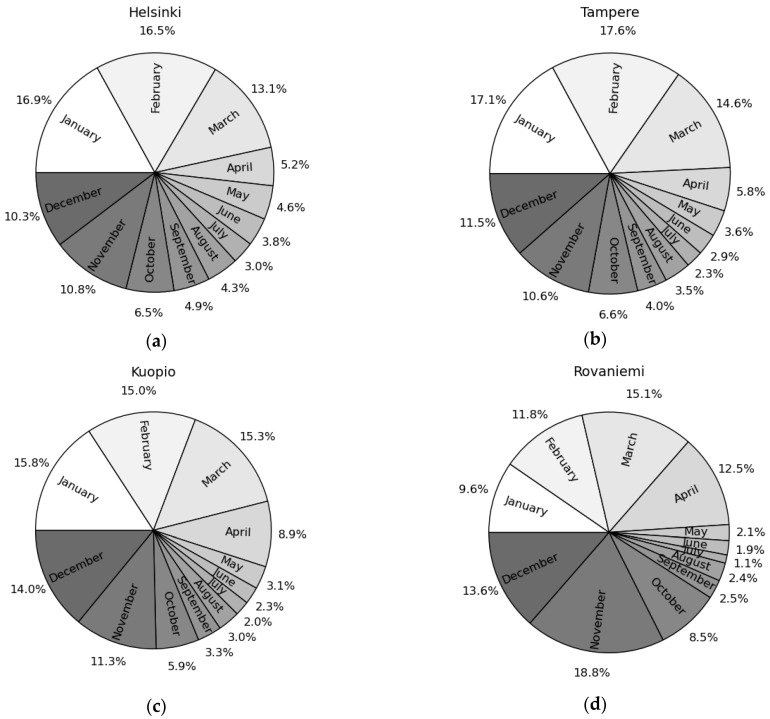
Pie charts showing the portion of each month’s slip injuries based on the TVK’s data for Helsinki (**a**), Tampere (**b**), Kuopio (**c**) and Rovaniemi (**d**) with a time range between 1 October 2005, and 30 September 2019. Different shades of gray present different months from January (white) to December (dark gray). Figures for all cities are presented in the Supplementary Material (Figure S1).

As can be expected, the total amount of slips is much higher during winter than summer. According to the TVK’s data, winter months January, February, and March cover about half of the yearly slip injuries except in eastern and northern part of Finland where the percentages for mid-winter months are slightly smaller (see Figure 2, the full set of the figures can be found from the Supplementary Material Figure S1).

When also considering the other winter months (April, October, November, and December) the percentage is between 80 and 90 (Table 1 and Figure 2). However, the total number of slips and falls in summer months (June–August) is affected by the summer holiday season that reduces the number of commuting accidents.

**Figure 3:**
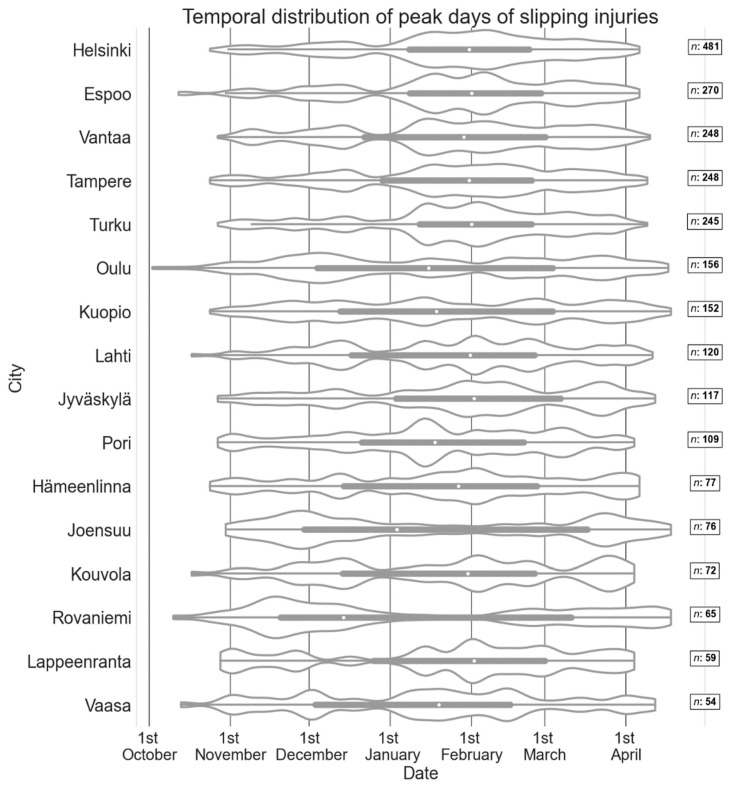
The temporal frequency distribution of peak days of slipping injuries in different cities in winters 2005–2019 presented on a violin plot. The width of the span corresponds to the frequency of slip injuries, the wider the distribution is the more slip injuries happen. The white dot within each box represents the median dates of the winter season slips. The box spans the 0.25 and 0.75 quantiles, and the whiskers represent the minimum and the maximum dates. Outliers are within the violin plot area. The number of peak days (*n*) is presented for each city on the right.

Figure 3 presents the temporal distribution of the peak days of slipping injuries in different cities. The peak days are presented as a function of sequential numbering of days starting from October 1. To make the figure easier to read, the sequential numbering of days has been changed to calendar days (i.e., 1st is October 1, 31st is November 1, and so on). In the case of leap years (2008, 2012 and 2016) there is a one-day shift on peak days occurring in March and April.

The beginning of the slip season and the first peak days of slip injuries occur typically in October, and the end of the season is in April. In south coastal cities, Helsinki, Espoo and Turku, the peak days are concentrated in the middle of the winter, with 50% of the peak days occurring within a shorter time period than elsewhere. Cities located in the east or north (Joensuu, Oulu and Rovaniemi) have more slips in the beginning and at the end of winter compared to mid-winter. This is strongly related to the climate because near zero temperatures are not frequent in the eastern and northern part of Finland in the middle of the winter, as can be seen in Figure S4 on Supplementary Material. In places where the slip season is usually short, the median of the slips is typically dated at the turn of January–February, whereas in other places the median is reached earlier. In Rovaniemi, the beginning of the slip season seems to be somehow more intensive with more slips than in other places. The holiday season, especially Christmas time and the New Year period are visible in the data with lower amounts of slips.

**Figure 4:**
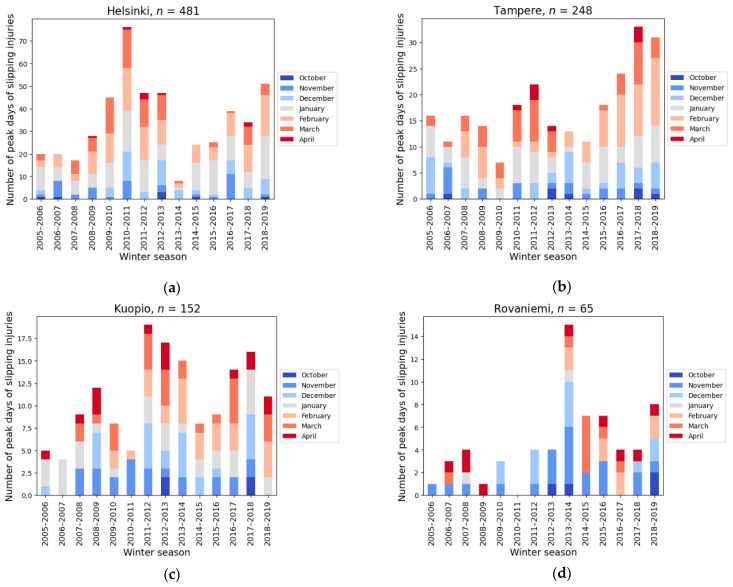
The monthly distributions of the peak days on different winters for Helsinki (**a**), Tampere (**b**), Kuopio (**c**) and Rovaniemi (**d**) between the years 2005 and 2019. The number of peak days (*n*) is shown for each city. Figures for all cities are presented in the Supplementary Material (Figure S2).

The monthly distribution of peak days for four of the cities is presented for all winter seasons (2005–2019) in Figure 4a–d (the full set of the figures can be found from the Supplementary Material Figure S2). The number of peak days per winter is the highest in Helsinki. The difference between winter seasons can be large but there can also be large differences within one winter season. For example, during winter season 2010–2011, there was a high number of peak days in Helsinki whereas during the same winter Rovaniemi recorded zero peak days. On the contrary, during the winter season 2013–2014, Helsinki had only a few peak days, while Rovaniemi during the same time had the largest number of peak days (see Figure 4a,d). The winter of 2010–2011 was very snowy already from the beginning of winter season, and high snow amounts were measured also in the southern part of Finland during the winter. The temperature was below average. In the winter of 2013–2014, the temperature was 2–4 degrees warmer than the average. In the southern part of Finland, the snowy season was very short, and sidewalks were not covered by ice and snow for a long time. Meanwhile, the northern part of Finland had more zero degree temperatures and icy surfaces than normally [40].

The beginning of the winter season and the first slippery days can be challenging for pedestrians. Wrong footwear, unexpected slipperiness, and the lack of preparedness for the slippery sidewalk condition easily increase the slip risk. Similarly, tourists may encounter snow and ice for the first time in their life and be in general unfamiliar and unprepared to the Nordic winter environment [41]. The beginning of the winter season with the first snow falls and temperatures below 0 °C is often very visible when looking up slip statistics [30]. The general level of slip rate increases rapidly when it snows for the first time and the temperature drops below zero degrees.

In the springtime, snowy and icy sidewalks begin to melt. Warm air masses and increased shortwave radiation can cause large temperature variations. Because of increased differences between daytime and nighttime temperatures, it is possible that snow and ice on the sidewalks melt during the day and freeze again during the night, so sidewalk slipperiness can be quite different in the morning compared to the afternoon. It must be remembered that even when the snow has already melted from the area, the sidewalks and paths may be icy, especially in places that are in the shade.

### 3.3. Temperature on Peak Days of Slipping Injuries

**Figure 5:**
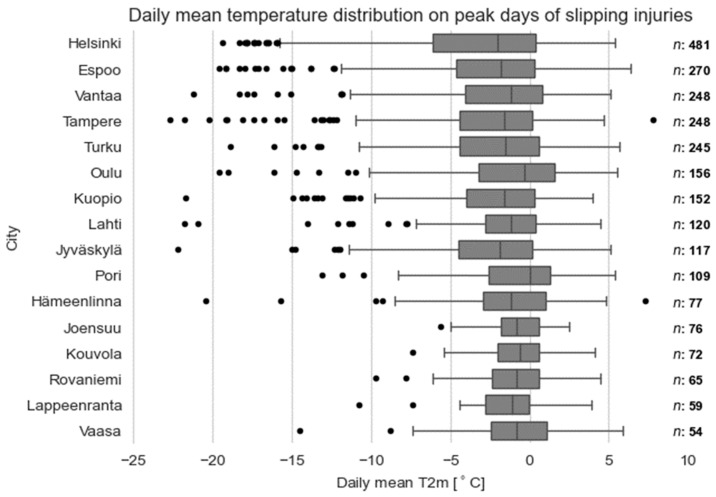
Box plot for daily mean temperature on peak days in different cities during the 14 winters between the years 2005 and 2019. Boxes correspond to upper and lower quartiles of the data, with the vertical line in each box showing the median. Whiskers indicate variability outside the quartiles and outliers are shown as individual black points. The number of peak days (*n*) for each city is shown on the right.

Near-zero temperatures or temperatures just below 0 °C can typically be seen to exist when peak days of slipping injuries occur. The statistics of the daily mean temperature on peak days of slip injuries are presented on box plots in Figure 5. The mean temperature of peak days varies between −3.3 °C (Helsinki) and −0.7 °C (Joensuu). The overall temperature scale of the peak days is quite large and there are several peak days when the daily mean temperature is very low, in some cases even below −20 °C. The temperature scale is larger in the bigger cities with a higher number of peak days compared to smaller cities with a lower number of peak days.

**Figure 6:**
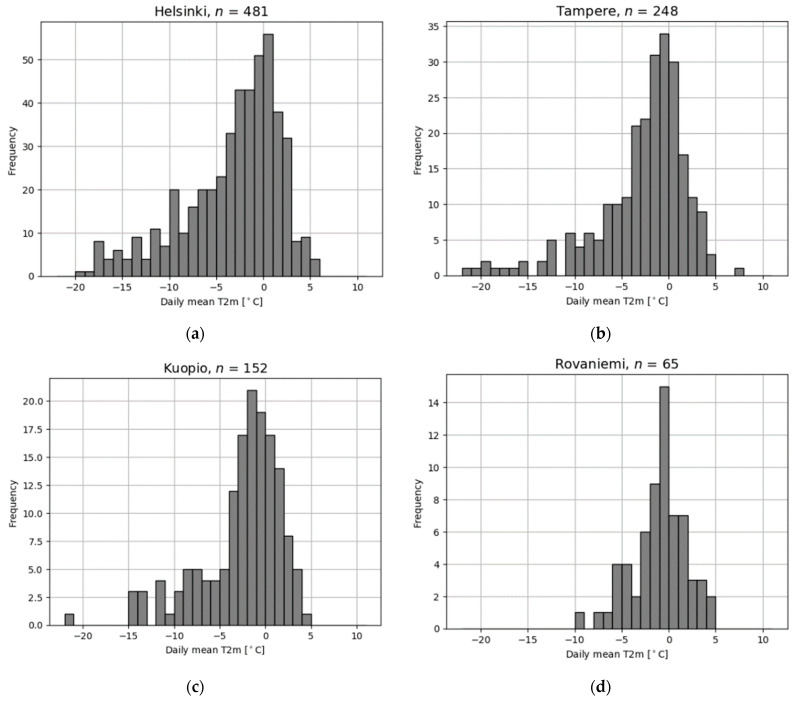
Frequency histograms of the daily mean temperature on peak days of slipping injuries for Helsinki (**a**), Tampere (**b**), Kuopio (**c**) and Rovaniemi (**d**) between the years 2005 and 2019. The number of peak days (*n*) is shown for each city. Figures for all cities are presented in the Supplementary Material (Figure S3).

Figure 6a–d show the frequency histogram for the daily mean temperature on the peak days for four of the cities (the full set of figures is presented in the Supplementary Figure S3). Typical daily mean temperature on the peak days is around 0 °C, with the mean temperature above zero more frequently in cities located on the coast than inland.

**Figure 7:**
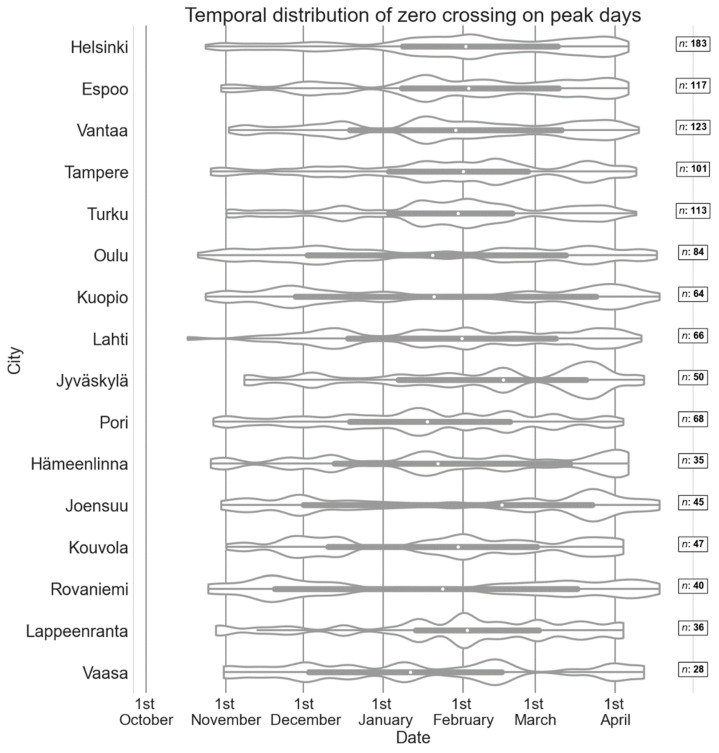
Temporal distribution of the wintertime zero crossings on peak days for all cities between October and April in winter 2005–2019 presented on a violin plot. The width of the span corresponds to the frequency of slip injuries, the wider the distribution is the more slip injuries happen. The white dot within each box represents the median dates of the zero crossings on the peak days. The box spans the 0.25 and 0.75 quantiles, and the whiskers represent the minimum and the maximum dates. Outliers are within the violin plot area. The number of zero crossing days during peak days (*n*) is presented for each city on the right.

Zero degree crossings cause slipperiness due to phase transition (from solid to liquid or vice versa) when moisture exists on the sidewalk surface. In this study, zero degree crossing cases are defined as days when daily maximum temperature is above 0 °C and minimum temperature below 0 °C. Temperature crossing 0 °C is most typical in springtime when the daily temperature fluctuation is at its largest (see Figure S4 on Supplementary Material). In the northern and eastern part of Finland zero crossings are not typical in mid-winter whereas in the other places, especially in the coastal cities, zero crossings happen throughout the winter. Similarly, in the northern and eastern part of the country zero crossings during peak days are not typical in mid-winter, but in many other cities they occur also in the mid-winter (Figure 7). The zero crossings on peak days are most typical in Kouvola (65.3%) and least typical in Helsinki (38.0%).

### 3.4. Precipitation and Snow Amount on Peak Days of Slipping Injuries

It is expected that precipitation raises the slip risk. Daily precipitation sum is recorded between 6 UTC on the date of the observation and 6 UTC on the following day. This measuring method brings some uncertainty to the results as the data is not available on a daily level. The precipitation form is also not available. According to the results, precipitation (daily precipitation sum 0.3 mm or more) on peak days of slip injuries varies between 50.7% (Helsinki) and 72.3% (Joensuu). Similarly, the percentages for precipitation sums of at least 2 mm vary from 30.1 (Helsinki) to 49.2 (Rovaniemi).

The impact of snow on the slip amounts is also investigated in this study. According to the weather and slip injury data, snow cover raises the number of slip injuries over three-fold and there are differences between the cities. When comparing snowy situations to no-snow situations, the ratio is lowest in Helsinki (3.2), and highest in Kouvola (5.5). Snowy situations are defined as days when the observed snow amount has been 1 cm or more (measurement done at 6 UTC). Measured snow cover doesn’t necessarily mean icy or snowy sidewalk conditions and vice versa but it is, nevertheless, the best data that is available to describe the snow and ice situation on the sidewalks.

It is found that in the case of bigger cities, the longer the snowy season, the higher the number of peak days per winter season. The correlation shows that the number of the peak days increases as a function of the length of the snowy season. In the case of smaller cities, however, the signal is weak or even opposite compared to bigger cities.

The day with the highest number of slip injuries was 24 November 2008, in Helsinki, Espoo and Vantaa. Snow accumulation was almost 30 cm during that day and on the day before, and temperature was about −1 °C. The reasons for the high slip injury numbers can only be speculated. The situation was favorable for the slipperiness due to packed snow that occurs when the temperature is just below 0 °C, it is snowing, and there are enough people walking to compress the snow. Most probably, the snow removal crew was busy because of continuous and long lasting snowfall and there were challenges to maintain the sidewalks properly. Large amounts of snow also create barriers on the walking routes which can raise the slip risk. In addition, the situation was one of the first actual slippery days for that winter season, so people weren’t necessarily yet prepared for slippery sidewalk conditions. High slip amounts were also recorded over the next three days, maybe due to high snow amounts but also because the temperature rose above 0 °C.

### 3.5. Weather before Peak Days of Slipping Injuries

The weather on the previous days (1–3) can play a significant role in the slipperiness of the following days. If temperature drops below zero degrees, moist surfaces freezes. Frozen surfaces can be slippery themselves but even a light snowfall can make surfaces more slippery still if the light and loose snow covers the hard and smooth ice layer. Pedestrians don’t necessarily notice the risk of slipperiness as the icy surface is covered by snow [30]. Also, lots of new snow may cause slipperiness for several days if the winter road maintenance is unable to reduce sidewalk slipperiness and remove the snow, as was noticed in case 28 November 2008.

In some cases, the slipperiness may also occur when the daily mean temperature is low, below −10 °C or even below −20 °C. Part of those cases can be explained by a notable temperature decrease during one or more days of continuing light snowfall. However, there are several cases with high slip rate also when precipitation has not been measured. In those cases, high slip rates can be due to hard ice layers or to other non-meteorological reasons. The number of these cases is quite small in the studied material, however, and the cases appear typically during mid-winter with high amounts of snow.

## 4. Discussion

Winters with ice, snow and near zero temperatures have a strong impact on sidewalk slipperiness and the findings of this research are consistent with the previous studies. According to the results, the mean temperature on peak days of slipping injuries is typically around zero or slightly below zero degrees. However, the temperature scale on peak days is large and there are also a few peak days when daily mean temperature is even below −20 °C. Also, zero-degree crossings are very typical on peak days and their occurrence is around 50% in all the cities studied here.

Precipitation (daily accumulation 0.3 mm or more) occurred on more than half of the peak days. Also, the length of the winter (snowy season) seems to correlate with the number of peak days of slips, especially in the bigger cities: the longer the snowy season is, the more peak days of slips seem to occur. In addition, lots of new snow in a short time period seems to increase the slip risk.

However, there are some cases when the high slip rate cannot be explained by weather. Non-meteorological factors, such as the lack of winter maintenance of sidewalks or the number of pedestrians making sidewalks slippery due to packed snow, can also influence slip rates. Also, weather on previous days may be the reason for slipperiness. Slipperiness is a very complex phenomenon, and further studies are needed to understand the role of non-meteorological factors affecting the risk of slips.

Helsinki, with the highest number of pedestrians, seems to have more non-weather-related issues affecting slip-injury rate than other cities. The use of sustainable transport modes, including walking, is more common in the Helsinki metropolitan area than in smaller cities [42]. This may explain at least partly why slips and slip-related injuries are more common especially in Helsinki compared to smaller cities.

It is expected that climate change will bring changes on slipperiness in Finland in the future. As wintertime temperatures are forecasted to rise, less icy and snowy sidewalk conditions in the beginning and at the end of the winter season can be expected because of increased occurrence of temperatures above or close to 0 °C. Season with ice and snow on the sidewalks will become shorter. However, the coldest places in Finland, especially Rovaniemi, Kuopio and Joensuu, may face the largest impacts due to global warming, because near-zero temperatures are becoming more frequent in the mid-winter [43], increasing slipperiness and risk of slips between January and March.

According to pedestrians themselves, the most important measures to prevent slips are the pedestrians’ own actions, like caution and the choice of footwear with good grip, as well as improvement in the winter maintenance of sidewalks [15]. The importance of winter maintenance has been highlighted in several studies [3,24]. Pedestrians’ awareness about slipperiness can be increased by warning about the expected slippery conditions. In addition to Finland, special warning services to inform citizens about slippery sidewalk conditions and the high risk of slips have been developed in Japan and Canada [30,44,45,46]. One method to improve safe walking is the use of anti-slip devices on a person’s ordinary shoes [47,48,49].

Instead of daily values, more detailed weather observations, for example hourly or more frequent data and precipitation form, would be beneficial when determining the state of slipperiness. Daily mean temperature observations are not always the best parameter to be used, especially in cases when the daily maximum temperature reaches above zero degrees during a day and the mean temperature stays below zero degrees. There are similar problems also in case of large temperature fluctuations during a day, or if air is cooling or warming. Also, surface temperature and the knowledge about existence of ice or snow on the sidewalk surface could give valuable information about potential slipperiness.

At present, there does not exist a measurement device that could measure operationally the sidewalk condition or slipperiness [30]. With a proper observation system, the state of slipperiness could be detected, which would help to estimate the forthcoming slipperiness. A proper and dense observation network would also give valuable information for winter maintenance.

Machine learning methods could offer more detailed information about the level of slipperiness and slip risk. Spatio-temporal prediction could consider the differences between cities, like climatology and number of pedestrians, and improve the prediction of high slip risk. Further research could also take into account additional information about slip injuries, including the type and severity of the injury, as well as and socio-demographic characteristics. A more detailed analysis of the cause-consequence relationships of slip amounts and weather could also be performed.

## 5. Conclusions

The aim of this study was to find out how the pedestrian slip rates vary between different seasons and months, and what is the weather typically like when high slip rates occur. Wintertime slipperiness due to ice and snow together with near zero temperatures clearly increases the risk of slips. Although temperatures are forecasted to rise in the future, slipperiness will still occur. As sustainable transport modes, especially walking, are becoming more recommended, it is important to invest in safety. The slip risk can be reduced by effective and timely winter sidewalk maintenance, the awareness of slippery sidewalk condition and risks of slipping, as well as by the use of the right footwear with good grip or anti-slip devices. Also, improvements in the coverage of slip-and-fall accident statistics are recommended. Additionally, traffic safety work should be broadened to cover single pedestrian accidents [50].

The Finnish national transport policy targets include increasing the combined share of walking and cycling and decreasing total transport emissions [51]. The promotion of walking and cycling helps to obtain the national health objectives, too. As the aim in the future is to increase the amount of walking, sidewalk condition forecasting, pedestrian awareness as well as winter maintenance should be improved. This could help to decrease the number of slips that cause personal suffering and also significant costs to society. Because the risk of slipping is highest where there is a lot of walking, the greatest benefits could be achieved in big cities where the number of pedestrians is high.

## Figures and Tables

**Table 1:** Information of the cities including the number of employees [37], amounts of slips, injury rate in winter, number of days with high slip rate and thresholds for peak day and potential peak day during 2005–2019 based on the TVK’s slip injury data.

City	Number of Employees	Total Amount of Slip Injuries	Total Amount of Slip Injuries in Winter	Injuries per Winter (%)	Injury Rate in Winter per 1000 Employees	Number of Days with High Slip Risk	Peak Day/Potential Peak Day Limit
Helsinki	413,677	38,627	30,658	79.4%	5.3	481	25/18
Espoo	126,820	10,376	8635	83.2%	4.9	270	12/8
Vantaa	122,871	10,014	8405	83.9%	4.9	248	12/8
Tampere	126,687	8878	7433	83.7%	4.2	248	11/7
Turku	105,364	8148	6898	84.7%	4.7	245	9/6
Oulu	92,267	5513	4887	88.6%	3.8	156	11/7
Kuopio	53,394	4148	3576	86.2%	4.8	152	8/5
Lahti	51,485	3533	3049	86.3%	4.2	120	7/5
Jyväskylä	64,937	3916	3417	87.3%	3.8	117	8/5
Pori	34,375	2408	2122	88.1%	4.4	109	6/4
Hämeenlinna	28,520	1829	1598	87.4%	4.0	77	6/4
Joensuu	34,321	1943	1738	89.4%	3.6	76	6/4
Kouvola	30,500	1797	1628	90.6%	3.8	72	6/4
Rovaniemi	27,311	1885	1694	89.9%	4.4	65	6/4
Lappeenranta	31,700	1799	1591	88.4%	3.6	59	6/4
Vaasa	37,070	1682	1491	88.6%	2.9	54	6/4

## Data Availability

Daily slip injury data was obtained from Finnish Workers’ Compensation Centre and are available from the authors with the permission of Finnish Workers’ Compensation Centre. Publicly available gridded weather observations datasets were used in this study. This data can be found here: https://paituli.csc.fi (accessed on 1 March 2022). Publicly available air quality datasets can be found here: https://en.ilmatieteenlaitos.fi/download-observations (accessed on 1 March 2022).

## Supplementary Materials

The following supporting information can be downloaded at: https://www.mdpi.com/article/10.3390/ijerph19053007/s1, Figure S1: Pie charts showing the portion of each month’s slip injuries based on TVK’s data. Data covers 16 cities with a time range between 1 October 2005, and 30 September 2019. Figure S2: The monthly distribution of the peak days on different winters for all cities between the years 2005 and 2019. Figure S3: Frequency histogram of the daily mean temperature on peak days of slipping injuries for all cities between the years 2005 and 2019. Figure S4: Temporal distribution of the zero-degree crossings in winter months between October and April in winter 2005–2019.
