# Supplementary material for: Impact of Weather on Pedestrians’ Slip Risk
Source: Int J Environ Res Public Health. 2022 Mar 4;19(5):3007. doi: 10.3390/ijerph19053007 (PMC8910018; doi:10.3390/ijerph19053007)
Supplement: Supplementary file 1 [file ijerph-19-03007-s001.zip › ijerph-1600042-supplementary.pdf]

International Journal of
Environmental Research and Public Health

MDPI

Supplementary Material

Impact of Weather on Pedestrians' Slip Risk

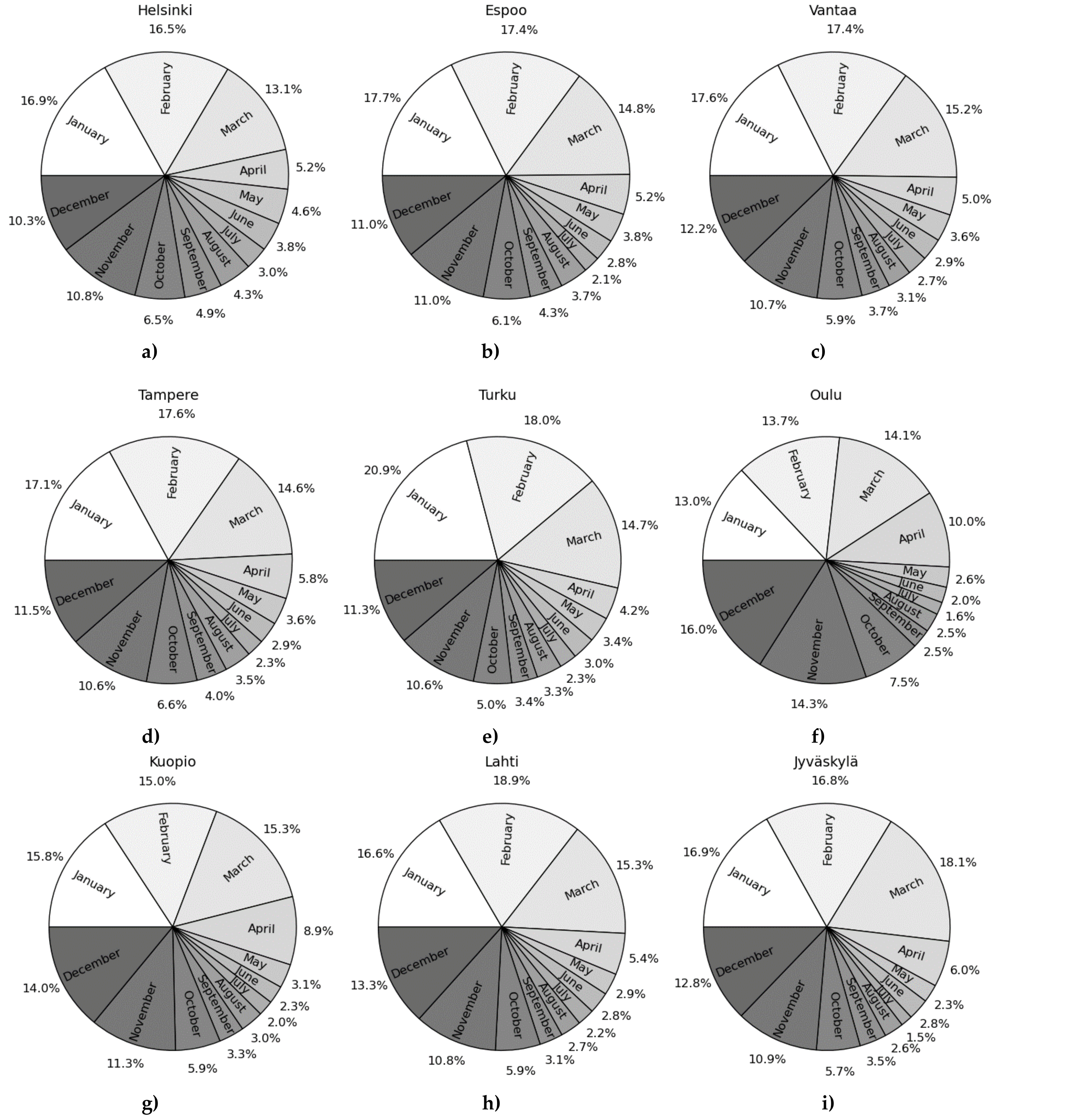

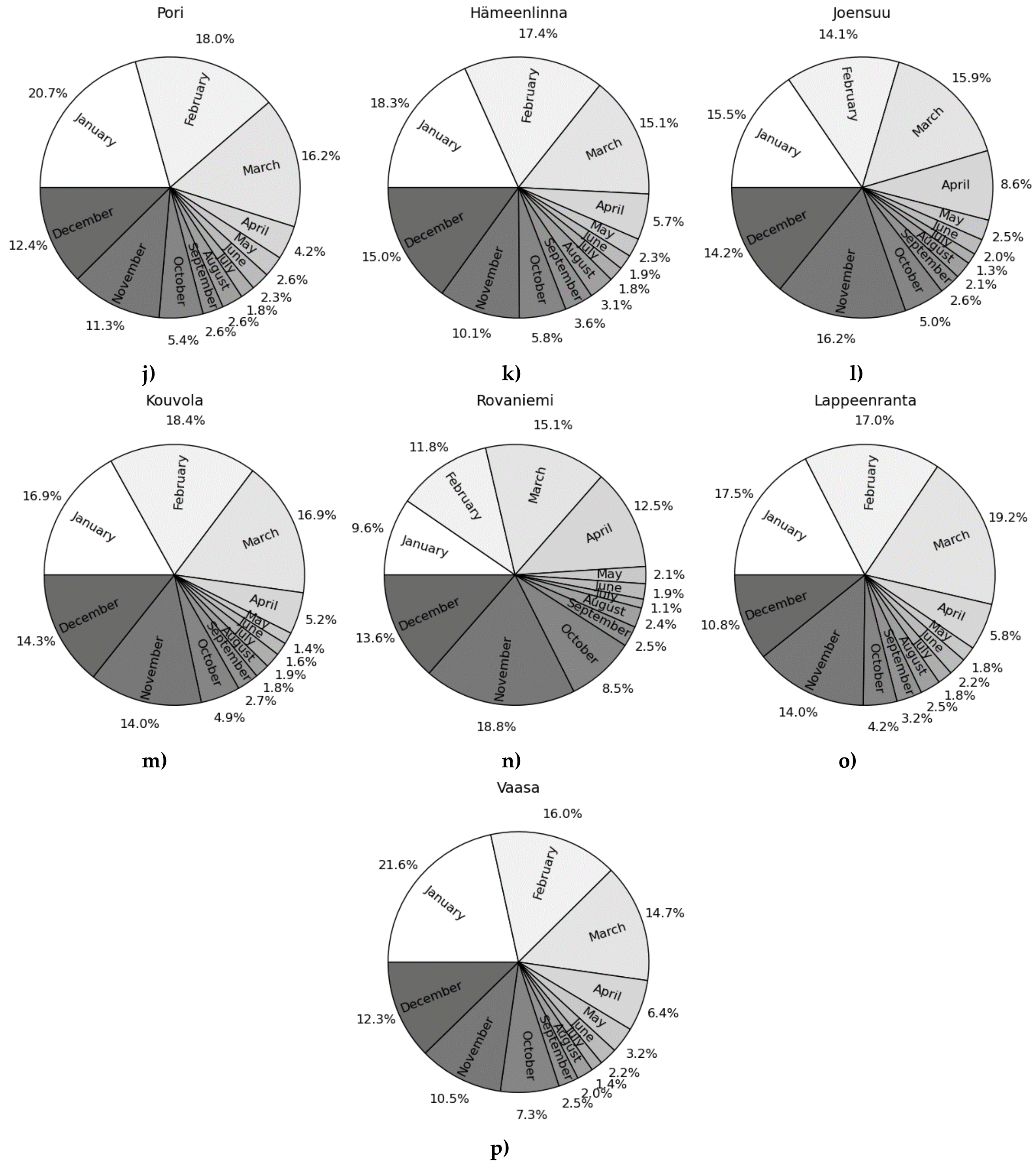

Figure S1. Pie charts showing the portion of each month's slip injuries based on TVK's data for all cities (a–p) with a time range between 1 October 2005, and 30 September 2019. Different shades of gray present different months from January (white) to December (dark gray).

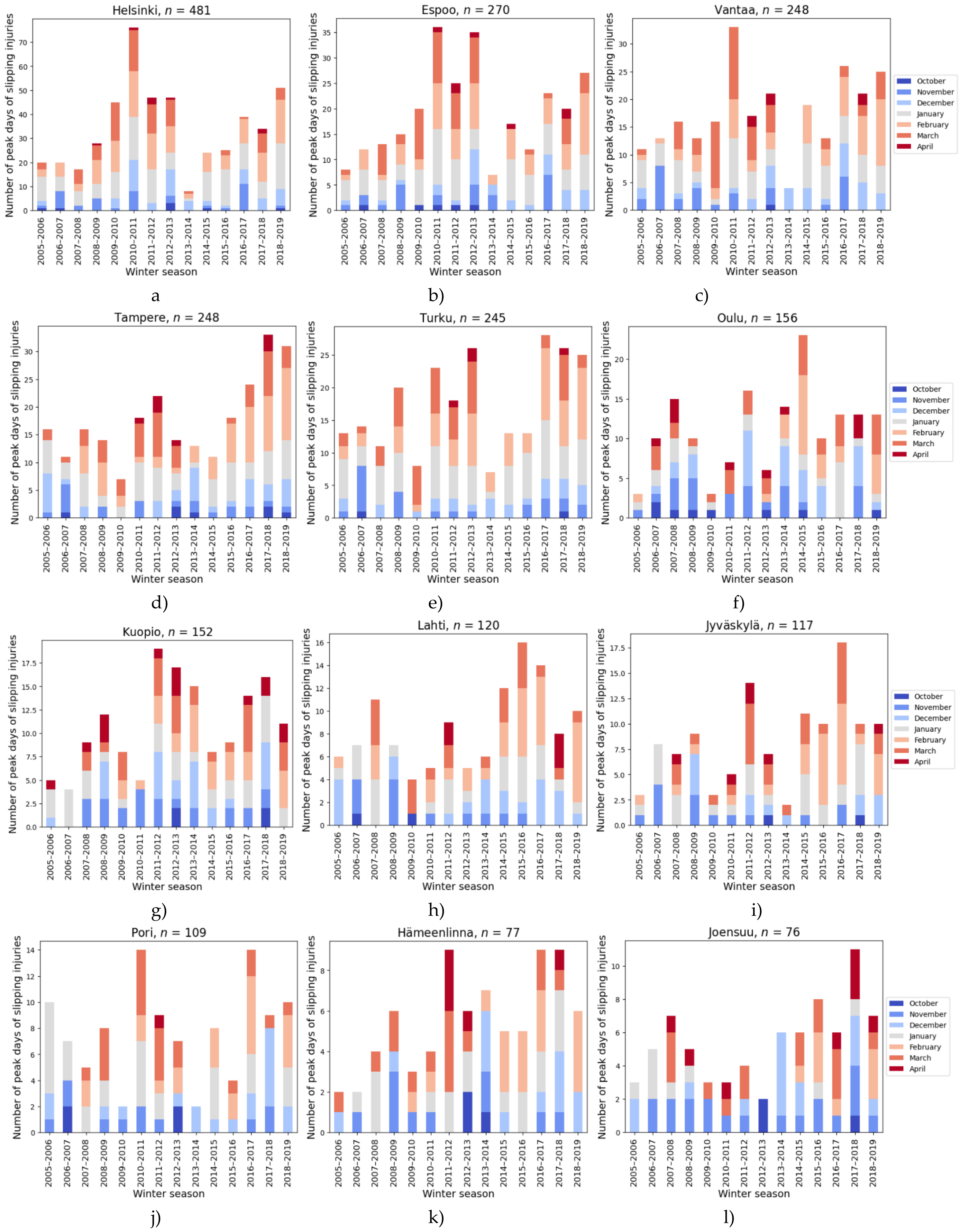
Helsinki, n = 481
Espoo, n = 270
Vantaa, n = 248
Tampere, n = 248
Turku, n = 245
Oulu, n = 156
Kuopio, n = 152
Lahti, n = 120
Jyväskylä, n = 117
Pori, n = 109
Hämeenlinna, n = 77
Joensuu, n = 76
Number of peak days of slipping injuries
Winter season
2005–2006
2006–2007
2007–2008
2008–2009
2009–2010
2010–2011
2011–2012
2012–2013
2013–2014
2014–2015
2015–2016
2016–2017
2017–2018
2018–2019
October
November
December
January
February
March
April
a
b)
c)
d)
e)
f)
g)
h)
i)
j)
k)
l)

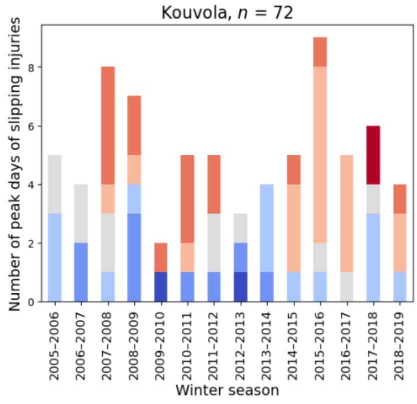

m)

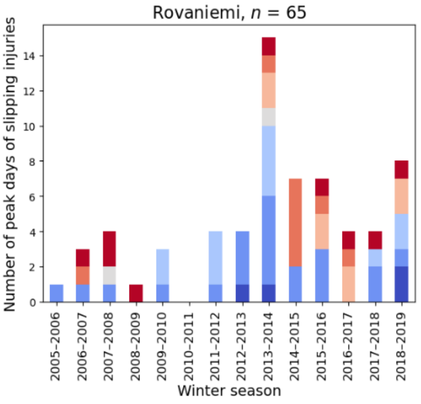

n)

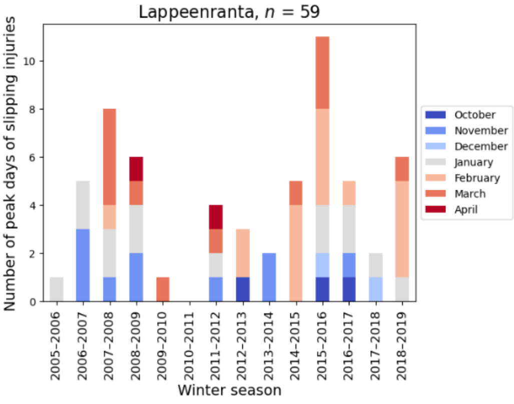

o)

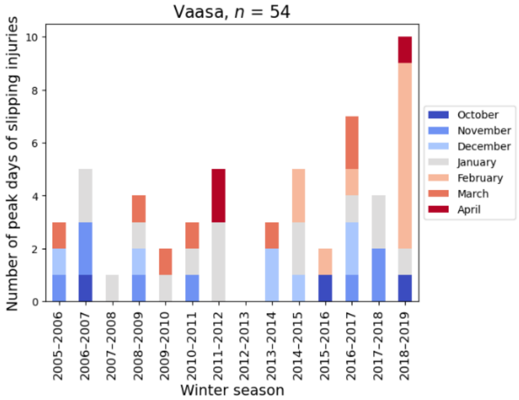

p)

Figure S2. The monthly distributions of the peak days on different winters for all cities (a–p) between the years 2005 and 2019. The number of peak days (*n*) is shown for each city.

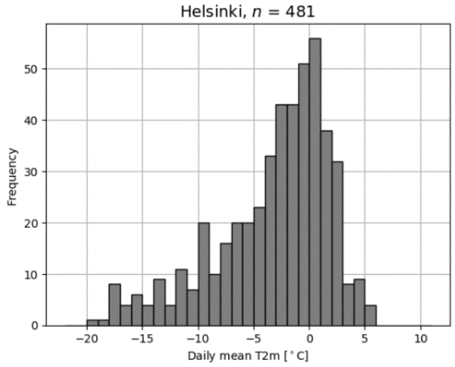

a)

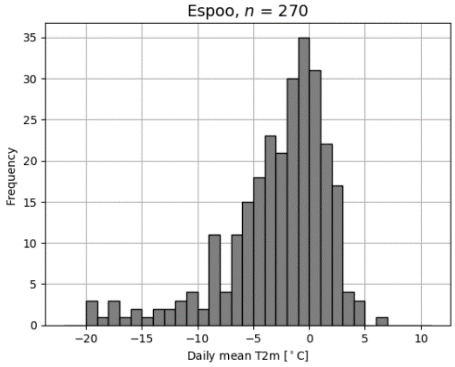

b)

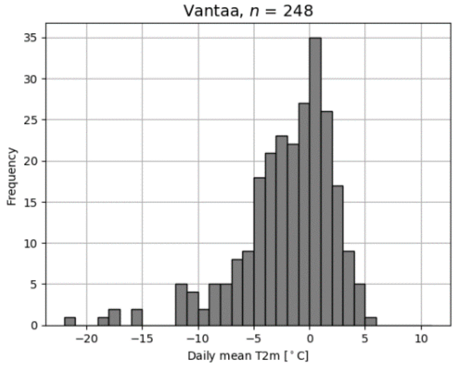

c)

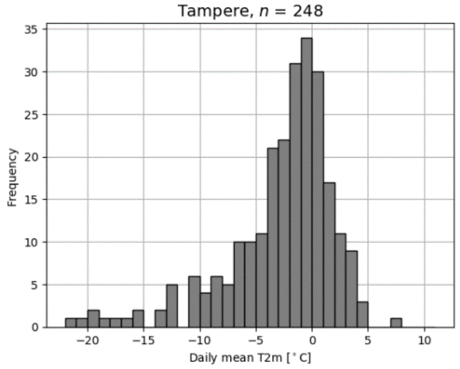

d)

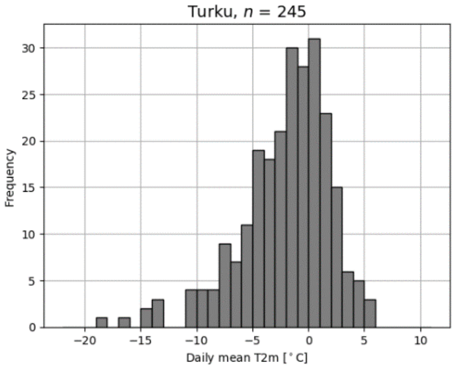

e)

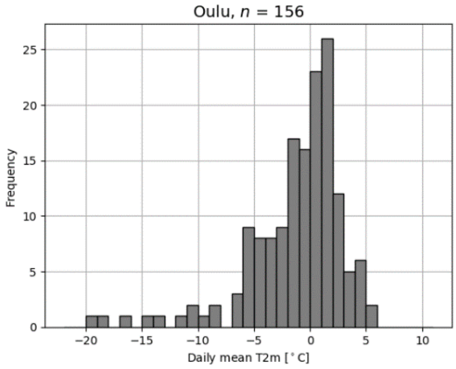

f)

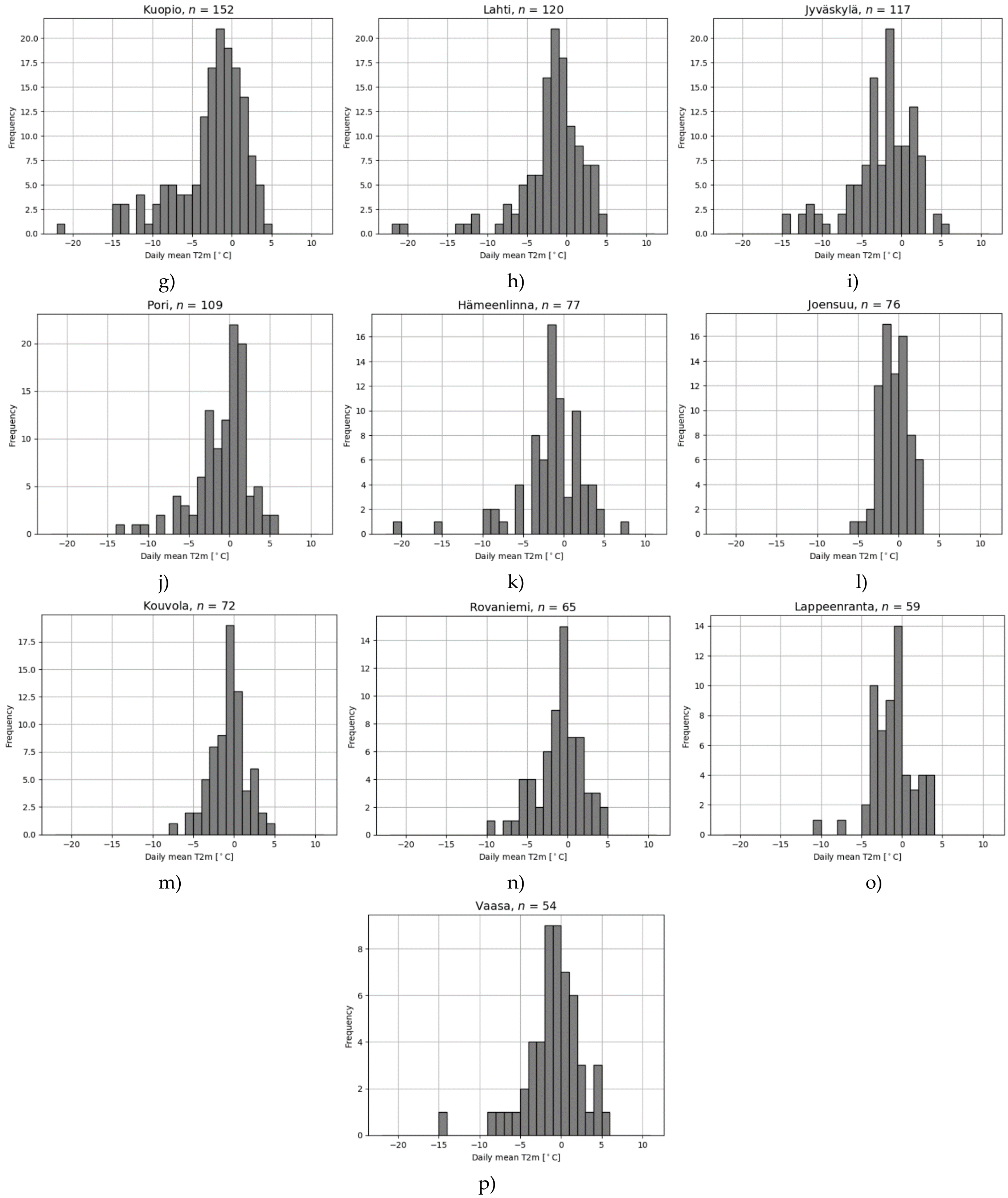

Figure S3. Frequency histograms of the daily mean temperature on peak days of slipping injuries for all cities (a–p) between the years 2005 and 2019. The number of peak days (n) is shown for each city.

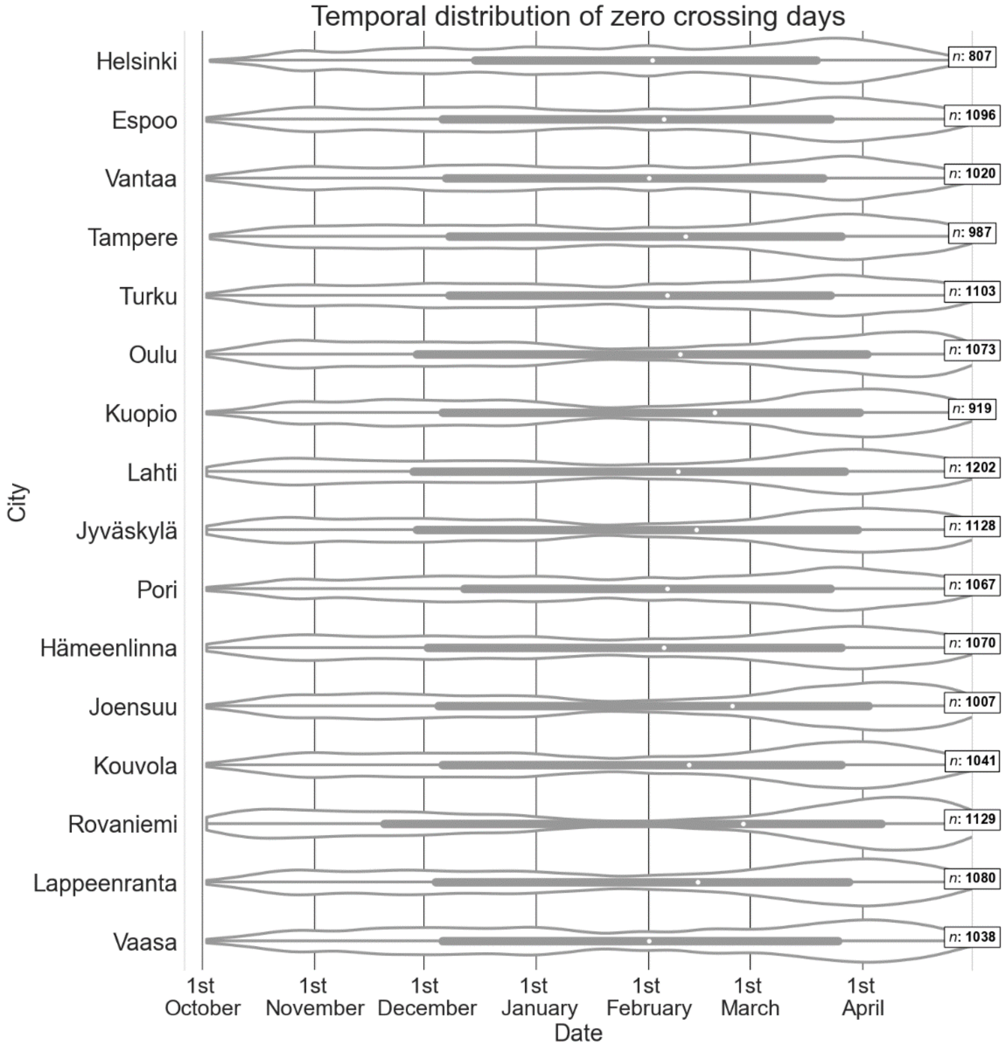

Figure S4. Temporal distribution of the zero-degree crossings in winter months for all cities between October and April in winter 2005–2019 presented on violin plot. The width of the span corresponds to the frequency of zero-degree crossings. The white dot within each box represents the median dates of the winter season zero-degree crossings. The box spans the 0.25 and 0.75 quantiles, and the whiskers represent the minimum and the maximum dates. The number of zero-degree crossing days (n) for the study period (October 2005–September 2019) is shown for each city on the right.
